# Nuclei-Guided Network for Breast Cancer Grading in HE-Stained Pathological Images †

**DOI:** 10.3390/s22114061

**Published:** 2022-05-27

**Authors:** Rui Yan, Fei Ren, Jintao Li, Xiaosong Rao, Zhilong Lv, Chunhou Zheng, Fa Zhang

**Affiliations:** 1 High Performance Computer Research Center, Institute of Computing Technology, Chinese Academy of Sciences, Beijing 100045, China; yanrui20b@ict.ac.cn (R.Y.); renfei@ict.ac.cn (F.R.); jtli@ict.ac.cn (J.L.); lvzhilong17g@ict.ac.cn (Z.L.); 2 University of Chinese Academy of Sciences, Beijing 101408, China; 3 Department of Pathology, Boao Evergrande International Hospital, Qionghai 571435, China; raoxiaosong2006@126.com; 4 Department of Pathology, Peking University International Hospital, Beijing 100084, China; 5 College of Computer Science and Technology, Anhui University, Hefei 230093, China; zhengch99@ahu.edu.cn

**Keywords:** breast cancer grading, histopathological image, nuclei segmentation, convolutional neural network, attention mechanism

## Abstract

Breast cancer grading methods based on hematoxylin-eosin (HE) stained pathological images can be summarized into two categories. The first category is to directly extract the pathological image features for breast cancer grading. However, unlike the coarse-grained problem of breast cancer classification, breast cancer grading is a fine-grained classification problem, so general methods cannot achieve satisfactory results. The second category is to apply the three evaluation criteria of the Nottingham Grading System (NGS) separately, and then integrate the results of the three criteria to obtain the final grading result. However, NGS is only a semiquantitative evaluation method, and there may be far more image features related to breast cancer grading. In this paper, we proposed a Nuclei-Guided Network (NGNet) for breast invasive ductal carcinoma (IDC) grading in pathological images. The proposed nuclei-guided attention module plays the role of nucleus attention, so as to learn more nuclei-related feature representations for breast IDC grading. In addition, the proposed nuclei-guided fusion module in the fusion process of different branches can further enable the network to focus on learning nuclei-related features. Overall, under the guidance of nuclei-related features, the entire NGNet can learn more fine-grained features for breast IDC grading. The experimental results show that the performance of the proposed method is better than that of state-of-the-art method. In addition, we released a well-labeled dataset with 3644 pathological images for breast IDC grading. This dataset is currently the largest publicly available breast IDC grading dataset and can serve as a benchmark to facilitate a broader study of breast IDC grading.

## 1. Introduction

Breast invasive ductal carcinoma (IDC) is the most widespread type of breast cancer, making up approximately 80% of all diagnosed cases. Histological grading has direct guiding significance for the prognostic evaluation of IDC. The most popular grading scheme is the Nottingham Grading System (NGS) [1] which gives a more objective assessment than previous grading systems. NGS includes three semi-quantitative criteria: mitotic count, nucleus atypia, and tubular formation. However, in clinical practice, the burden of pathological diagnosis is very heavy, and many pathologists cannot accurately grasp NGS, which will greatly weaken the guiding significance of histological grading for clinical prognosis evaluation, and even mislead the clinical judgment of prognoses. Therefore, there is an urgent need for an automatic and accurate pathological grading method.

The automatic breast cancer grading methods based on pathological images can be summarized into two categories. The first category is to use machine-learning or deep-learning methods to directly extract the features of the pathological image for breast cancer grading. However, unlike the coarse-grained problem of breast cancer classification, IDC grading is a fine-grained classification problem. Using only general methods cannot classify IDC well because the classification boundaries among intermediate-grade and low- and high-grade IDC pathological images are blurred.

The second category is to compute the three evaluation criteria of NGS separately and then integrate those results to obtain the final IDC grading result. However, NGS is only a semiquantitative evaluation method. The inherent medical motivation of NGS is to classify IDC based on the morphological and texture characteristics of the cell nucleus and the topological structure of the cell population. With the end-to-end advantage of deep learning, not only can the medical goal of emphasizing nuclei-related features be achieved, but more fine-grained feature representations of pathological images that are too abstract for pathologists to understand can also be learned.

In this paper, we propose a Nuclei-Guided Network (NGNet) for IDC grading in hematoxylin-eosin (HE) stained pathological images. Specifically, our network includes two branches. The main branch is used to extract the feature representation of the entire pathological image, and the nuclei branch is used to extract the feature representation of the nuclei image. Then, the nuclei-guided attention module between the two branches plays the role of nucleus attention in end-to-end learning, so that more nuclei-related feature representations for IDC grading can be learned. In addition, the proposed nuclei-guided fusion module in the fusion process of two branches can further enable the network to focus on learning nuclei-related features. Overall, under the guidance of nuclei-related features, the entire NGNet can learn more fine-grained features for breast IDC grading. It should be pointed out that this is different from the general attention mechanism [2,3,4] that cannot artificially emphasize the region of interest.

Experimental results show that the proposed NGNet significantly outperforms the state-of-the-art method, achieving 93.4% average classification accuracy and 0.93 AUC with our released dataset. In addition, we release a new dataset containing 3644 pathological images with different magnifications (20× and 40×) for evaluating the IDC grading methods. Compared with the previous publicly available breast cancer grading dataset with only 300 images in total, the number of images in our dataset has increased by an order of magnitude. The dataset is publicly available from https://github.com/YANRUI121/Breast-cancer-grading (accessed on 1 April 2022).

## 2. Related Works

Recently, the application of deep learning has enabled breast cancer pathological image classification to achieve high performance. However, breast cancer classification is not enough for the final medical diagnosis. The classification must be subdivided and accurate to the extent of the pathological grade of the cancer, because the gold standard of the final medical diagnosis, the choice of treatment plan and the prediction of patient outcome are all based on the results of the pathological grade.

**Figure 1:**
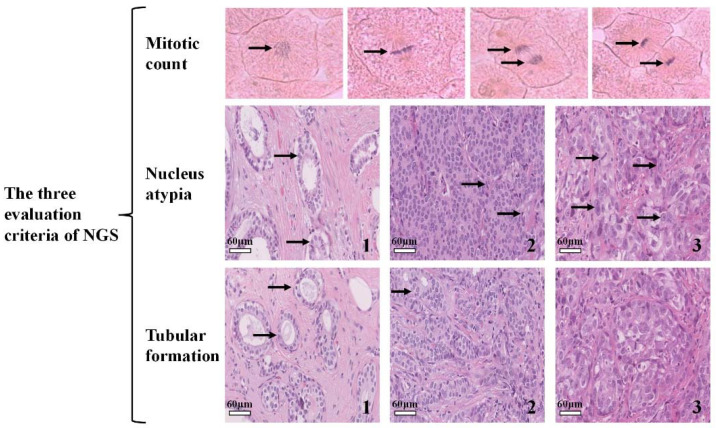
A brief description of the three evaluation criteria of NGS adopted by the World Health Organization. (1) Mitotic count: the images represent prophase, metaphase, anaphase and telophase stages of mitosis from left to right. (2) Nucleus atypia: the nucleus atypia score reflects the variations in the size, shape, and appearance of the cancer cells relative to normal cells. The nuclear atypia score values are 1, 2, and 3 from left to right. (3) Tubular formation: a large number of tubules are formed in the pathological image on the left. As the grade increases, the tubules gradually disappear from left to right.

The classification boundaries among intermediate-grade and low- and high-grade IDC pathological images are ambiguous; thus, general methods cannot classify the IDC grade well. The current IDC grading methods can be divided into two categories. The first category is to classify the features extracted directly from the pathological image. The second category is to first calculate the three evaluation criteria of NGS (1) mitotic count [5,6,7,8], (2) nucleus atypia [9,10], and (3) tubular formation [11,12,13], and then artificially integrate these three criteria to obtain the final result. Figure 1 is a brief description of NGS. By analyzing the three evaluation criteria of NGS, we observe that nuclei-related features are very important for breast cancer pathological diagnosis. Specifically, mitotic count and nucleus atypia are concerned with the morphological and texture characteristics of the cell nucleus, whereas tubular formation is concerned with the topological structure of the cell population. Because we are primarily concerned with end-to-end breast cancer grading studies, we will only briefly introduce the related works of the first category in the following.

Before the era of deep learning, research on breast cancer pathological image grading was mainly based on traditional machine-learning methods. For example, Doyle et al. [14] proposed a novel method to classify low- and high-grade of breast cancer histopathological images by using architectural features. Naik et al. [15] classify the low- and high-grade breast cancer by using a combination of low-level, high-level, and domain-specific information. They first segment glands and nuclei. Then, morphological and architectural attributes derived from the segmented gland and nuclei were used to discriminate low-grade from high-grade breast cancer. Basavanhally et al. [16] conducted a multifield-of-view classifier with robust feature selection for classifying ER+ breast cancer pathological images. Their grading system can distinguish low- vs. high-grade patients well, but fails to distinguish low- vs. intermediate-, and intermediate- vs. high-grade patients well.

Deep learning has made great progress in breast cancer pathological image grading. The most representative work was proposed by Wan et al. [17]. They integrated semantic-level features extracted from a convolutional neural network (CNN), pixel-level texture features, and object-level architecture features to classify low-, intermediate-, and high-grade breast cancer pathological images. The method achieved an accuracy of 0.92 for low vs. high, 0.77 for low vs. intermediate, and 0.76 for intermediate vs. high, and an overall accuracy of 0.69 when discriminating all three grades of breast cancer pathological images. Our preliminary work that shows that only using deep learning can help achieve better grading performance was published in BIBM2020 [18]. Compared to the previous work, we put forward new contributions in nuclei-guided branch fusion and further disclosed one of the largest IDC grading datasets.

In the field of computer vision, there are many excellent networks based on attention mechanisms, such as SENet [19], Position and Channel Attention [20], CBAM [4], Criss-Cross Attention [21], and Self-Attention [22,23]. SENet [19] is the abbreviation of Squeeze-and-Excitation Networks. SENet mainly recalibrates the feature responses of channels adaptively by explicitly modeling the interdependence between channels. In other words, the correlation between channels is learned. Convolutional Block Attention Module (CBAM) [4] combines spatial and channel attention mechanism, which can achieve better results than SENet’s attention mechanism that only focuses on channels. Because CBAM is a lightweight general module, it can be integrated into any CNN architecture with negligible overhead of this module, and can be trained end-to-end together with the base CNN. Transformer is a deep neural network based on self-attention mechanism, which has been considered as a viable alternative to convolutional and recurrent neural networks. In the field of computer vision, Vision Transformer (ViT) proposed by Dosovitskiy et al. [24] is a pioneering work. Following the paradigm of ViT, a series of ViT variants [25,26] have been proposed to improve the performance. The complexity of the ViT-like model is very high, so it needs a very large training dataset. Therefore, the application of the ViT-like model in the field of pathological images analysis is still few at present, especially for the breast cancer grading tasks that are difficult to manually label. These above-mentioned attention mechanisms are adaptively learned from the data, and are the areas where the algorithm thinks attention should be focused. However, if we need to customize the area where the algorithm focuses attention based on prior knowledge, this is not possible. A more comprehensive review of attention mechanisms can be found in [27,28]. Our proposed network can focus on a specific area. This is different from the general attention mechanism that cannot artificially emphasize the region of interest. This provides a new paradigm for embedding medical prior knowledge into algorithms.

## 3. Dataset

Deep-learning methods have an important dependence on well-labeled datasets such as BreaKHis dataset [29], the Yan et al. dataset [30], and the BACH dataset [31]. However, due to the difficulty of the IDC grading task, there are few related works. To the best of our knowledge, only Kosmas et al. [32] has released one IDC grading dataset containing 300 pathological images, which is insufficient for deep-learning research. In this work, we cooperated with Peking University International Hospital to release a new benchmark dataset for IDC grading. We conducted experiments on these two datasets to comprehensively verify the effectiveness of our proposed NGNet method. Next, we will introduce these two datasets.

### 3.1. IDC Pathological Images Dataset

The dataset released by Kosmas et al. [32] includes 300 images (107 Grade1 images, 102 Grade2 images, and 91 Grade3 images). All images were acquired at 40× magnification. Although this released dataset has played a significant role in the IDC grading research, 300 images are not enough for the deep-learning method.

**Figure 2:**
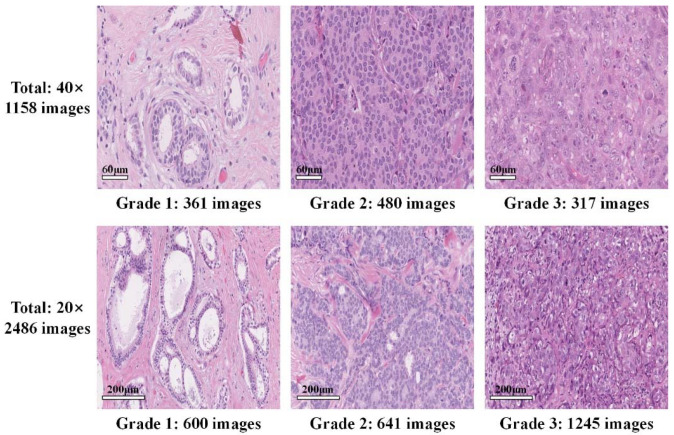
Pathological image examples and quantity statistics of our proposed dataset for IDC grading.

To meet the needs of deep-learning research, we cooperated with Peking University International Hospital to release a new IDC grading dataset. Our annotated HE-stained pathological image dataset consists of 3644 pathological images (1000 × 1000 pixels). Figure 2 is an example of the images and a summary of the dataset. We named it the PathoIDCG dataset, which is an abbreviation of the Pathological Image Dataset for Invasive Ductal Carcinoma Grading. The overall description of the PathoIDCG dataset is shown in Table 1. The preparation procedure used in our research is the standard paraffin process, which is widely used in routine clinical practice. The thickness of pathological sections is 3–5 μm. Each image is labeled Grade1, Grade2, or Grade3 according to the three evaluation criteria of NGS. Image annotation was independently performed by two pathologists in strict accordance with NGS standards, and the images with different annotations were reannotated by a senior pathologist. The Ethics Committee of Peking University International Hospital reviewed and approved the study, and all the related data are anonymous.

Our dataset is mainly acquired under a 20× magnified field of view, because the 20× magnified pathological image can contain more information about the topology of the cell population. Another reason is that the commonly available 20× slides are easier to obtain, and the current cell nucleus segmentation technology can also segment pathological images under 20× magnification. At the same time, we also collected pathological images at 40× magnification because a larger magnification can better reflect the texture and morphological characteristics of individual nuclei.

### 3.2. Nuclei Segmentation Dataset

**Figure 3:**
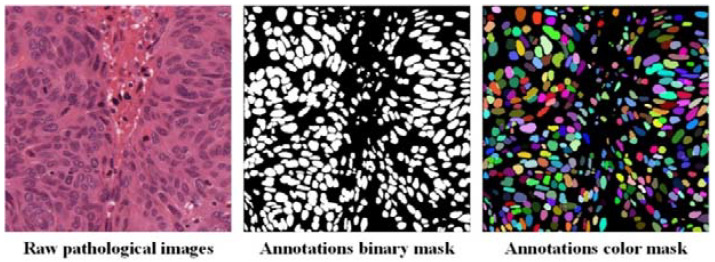
The nuclei segmentation dataset we used for breast cancer grading. We only need binary mask annotations to train the segmentation model. For better visualization, each nucleus is shown in a different color.

The dataset released by Kumar et al. [33] included HE-stained pathological images with 21,623 annotated nucleus boundaries, and Figure 3 is an example of this dataset. Kumar et al. [33] downloaded 30 whole slide pathological images of several organs from The Cancer Genomic Atlas (TCGA) [34] and used only one WSI per patient to maximize nuclear appearance variation. In addition, these images come from 18 different hospitals, which makes the dataset sufficiently diverse. It is important to emphasize that although we only segmented the nucleus of breast cancer pathological images, our segmentation model was trained on pathological images of all seven organs: breast, liver, colon, prostate, bladder, kidney, and stomach. For the above reasons, our segmentation model is more robust and generalizable.

## 4. Methods

**Figure 4:**
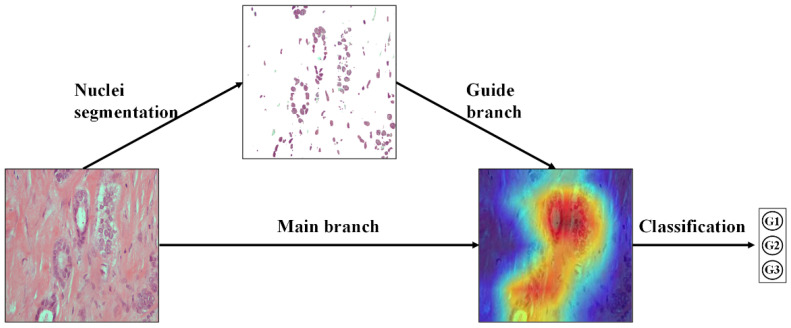
The key idea of the proposed method. NGNet forces the network to focus on learning features related to the nuclei. At the same time, under the guidance of nuclei-related features, the entire network learns more fine-grained features. The visual heat map is obtained through Grad-CAM using our proposed NGNet.

The key idea of NGNet is shown in Figure 4. Our method consists of two stages: in the first stage, we segmented the nucleus of each pathological image to obtain all images that only contain the nucleus region. In the second stage, two images (original pathological image and corresponding nuclei image) are input at the same time and sent to the NGNet to obtain the final classification result.

### 4.1. Nuclei Segmentation

We use DeepLabV3+ [35] as our nuclei segmentation network because it can better address the following challenges. In the HE-stained pathological image, some cell nuclei are very large, whereas some are very small. Moreover, under different magnifications, such as 20× and 40×, the difference in the size of the nucleus is more significant. Therefore, our network is required to be able to use multiscale image features, especially to be able to reconstruct the information of small objects. At the same time, many overlapping nuclei boundaries make nuclei segmentation more difficult, so the segmentation algorithm is required to have the ability to reconstruct nuclei boundaries.

Given a pathological image, the output of DeepLabV3+ is a nuclei segmentation mask. The backbone of the DeepLabV3+ algorithm we applied is Xception [36]. When our training steps are 100,000, we have achieved the best experimental results. The values of atrous rates we used are 6, 12, and 18. We adopt an output stride equal to 16. Here, we denote the output stride as the ratio of input image spatial resolution to the final output resolution.

### 4.2. NGNet Architecture

**Figure 5:**
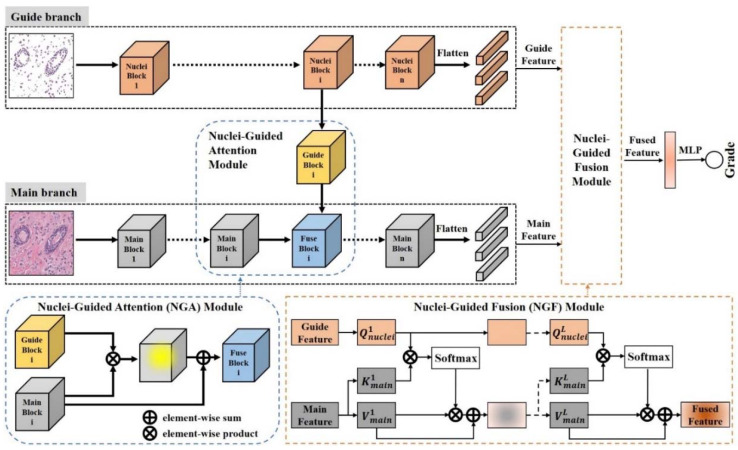
The overall network architecture of NGNet we proposed. The input of NGNet has two corresponding images: one is the original pathological image, and the other is the result of nucleus segmentation corresponding to this original pathological image. The entire NGNet is trained end-to-end.

The overall network architecture is shown in Figure 5. The proposed NGNet has two inputs [$I_{main},I_{nuclei}$]. The input to the main branch is the original pathological image $I_{main}$, and the input to the guide branch is the image $I_{nuclei}$ containing only the nuclei, respectively. The relationships between the two inputs are:

(1) $$I_{nuclei}=S\times I_{main},$$

where *S* is the nuclei segmentation result corresponding to the original pathological image.

The guide branch and main branch contain the same number of convolutional layers. Between the corresponding convolutional layers of the two branches, the Nuclei-Guided Attention (NGA) module transfers the nuclei-related features of the guide branch to the main branch. On top of the last convolution layer of each branch, feature maps $F_{main}^{M}\left(n\right)$ and $F_{nuclei}^{M}\left(n\right)$ were flattened to several feature vectors $P_{main}^{M}\left(n\right)$ and $P_{nuclei}^{M}\left(n\right)$, respectively, where *M* represents the number of convolutional layers of each branch, and *n* represents the *n*-th feature map. Then, the feature vectors $P_{main}^{M}\left(n\right)$ and $P_{nuclei}^{M}\left(n\right)$ were passed through the Nuclei-Guided Fusion (NGF) module to obtain fused feature representation. Finally, the grading result is obtained through the multilayer perceptron (MLP) module.

**Figure 6:**
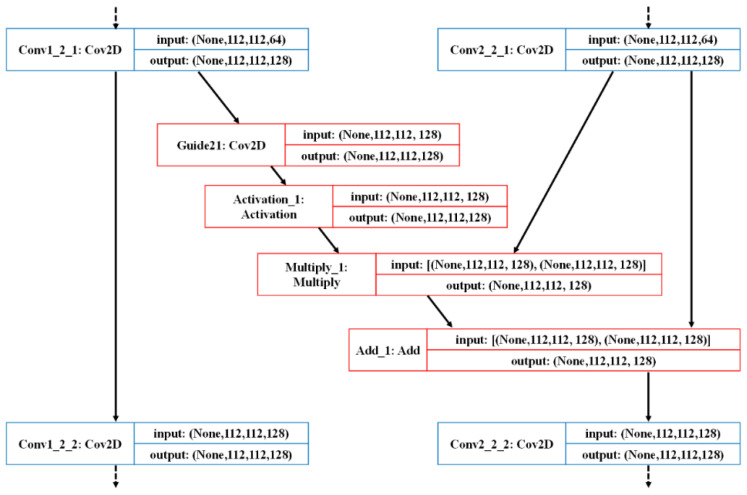
Detailed schematic diagram of the nuclei-guided attention module in the NGNet we proposed; the example comes from the “Guide 21” step.

The following is a detailed introduction to the NGA module and NGF module. The specific implementation details of the NGA module can be illustrated by the specific example of the “Guide 21” step in NGNet, as shown in Figure 6. Given a pathological image *I*, $F_{main}^{m}\left(I_{main}\right)$ and $F_{nuclei}^{m}\left(I_{nuclei}\right)$ is denoted as the convolutional feature maps from the *m*-th convolutional layer of the main branch and guide branch, respectively. In each corresponding convolutional layer, the guide branch extracting nuclei features has a guide block $F_{guide}^{m}\left(I_{nuclei}\right)$ pointing to the main branch extracting pathological image features.

We first perform a 1 × 1 convolution on the feature maps of the corresponding nuclei block $F_{nuclei}^{m}\left(I_{nuclei}\right)$, in which the input and output dimensions are equal. After performing the 1×1 convolution operation on the feature maps of the corresponding nuclei block $F_{nuclei}^{m}\left(I_{nuclei}\right)$, the Softmax activation function is used to generate the attention map $A^{m}$. Thus, the value of the feature map is adjusted to between 0 and 1. Then, we perform elementwise multiplication with the feature map of the corresponding main branch $F_{main}^{m}\left(I_{main}\right)$, thereby increasing the weight of the important area of the feature map. The purpose of this is to focus on the features related to the nuclei. Specifically, we calculate the attention map $A^{m}$ and guide block $F_{guide}^{m}\left(I_{nuclei}\right)$ as follows:

(2) $$A^{m}=\mathrm{Softmax}\left(\;(Conv_{1\times 1}\left(F_{nuclei}^{m}\left(I_{nuclei}\right)\right)\right),$$

(3) $$F_{guide}^{m}\left(I_{nuclei}\right)=F_{main}^{m}\left(I_{main}\right)⊗\;A^{m},$$

where the Softmax (.) is the Softmax activation function, Conv_1__×1_ (.) is a 1 × 1 convolution operation, ⊗ represents elementwise multiplication. At the end of each NGA module, an elementwise addition ⊕ is performed:

(4) $$F_{fuse}^{m}\left(I\right)=F_{main}^{m}\left(I_{main}\right)\;⊕\;F_{guide}^{m}\left(I_{nuclei}\right),$$

where $F_{fuse}^{m}\left(I\right)$ is the feature maps guided by nuclei-related features from the *m*-th convolutional layer.

The NGF module (see Figure 5) is inspired by the self-attention mechanism which can capture various dependencies within a sequence (e.g., short-range and long-range dependencies). The self-attention mechanism is implemented via the Query-Key-Value (*QKV*) model. Given a sequence and its packed matrix representations of *Q*, *K*, and *V*, the scaled dot-product attention is given by

(5) $$Att\left(Q,\;K,\;V\right)=\mathrm{Softmax}\left(\frac{QK^{T}}{\sqrt{d_{k}}}\right)V=AV,$$

where *d_k_* is the dimension of key, and *A* is often called the attention matrix which computes the similarity score of the *QK* pairs. Different from the standard self-attention *QKV* which comes from the same input sequence, our $Q_{nuclei}$ is the feature vector from the guide branch, and the $K_{main}$, $V_{main}$ are the feature vectors from the main branch. Therefore, the $Q_{nuclei}K_{main}$ similarity we calculated represents the similarity between the nuclear features and the original pathological image features. The similarity score of $Q_{nuclei}K_{main}$ is then mapped to $V_{main}$, allowing the network to pay more attention to the nuclei-related features. The $Q_{nuclei}^{l}K_{main}^{l}V_{main}^{l}$ calculation can be performed one or more times (*L*); here we set *L* = 3. In addition, we also added a residual connection between $V_{main}^{l}$ and $Att\left(Q_{nuclei}^{l},\;K_{main}^{l},\;V_{main}^{l}\right)$ to preserve the information of the main branch. At the end of the NGF module, we obtained the fused feature representation of the guide and main branch. Formally, we have

(6) $$P=V_{main}^{L}+Att\left(Q_{nuclei}^{L},\;K_{main}^{L},\;V_{main}^{L}\right).$$

To get the final classification result, *P* is flattened into the vector, and then goes through the fully connected layer. The loss function for NGNet is defined as the cross entropy (CE) loss:

(7) $$L_{CE}=-\frac{1}{m}\overset{z}{\underset{i=1}{\sum }}\overset{k}{\underset{k=1}{\sum }}q_{k}^{z}\log\left(p_{k}^{z}\right),$$

where $q_{k}^{z}$ and $p_{k}^{z}$ indicates the ground truth and prediction probability of the *z*-th image for *k*-th class.

It should be emphasized that our method is universal and can be easily generalized to another task that needs to emphasize a certain local area (such as a lesion) in the model. First, determine the image area of interest through prior knowledge and segment this area. Then, our algorithm framework can model this particular part of attention into the algorithm through end-to-end learning. The design of this network structure provides an end-to-end modeling methodology for custom attention.

## 5. Results and Discussion

In this section, we will evaluate the performance of NGNet. We randomly selected 80% of the dataset to train and validate the model, and the remaining 20% was used for testing. All experiments in this paper are finished on three NVIDIA GPUs by using the Keras framework with TensorFlow backend. We mainly use the average accuracy to evaluate the performance of NGNet. Apart from the average accuracy, the classification performance of an algorithm can be further evaluated by using the sensitivity, specificity, confusion matrix, and AUC. The accuracy, sensitivity, and specificity metrics can be defined as follows:

$$\mathrm{Accuracy}=\frac{\mathrm{TP}+\;\mathrm{TN}}{\mathrm{TP}\;+\;\mathrm{TN}\;+\;\mathrm{FP}\;+\;\mathrm{FN}},$$

$$\mathrm{Sensitivity}=\frac{\mathrm{TP}}{\mathrm{TP}\;+\;\mathrm{FN}},$$

$$\mathrm{Specificity}=\frac{\mathrm{TN}}{\mathrm{TN}\;+\;\mathrm{FP}},$$

where TP (TN) represents number of true positive (true negative) classified pathological images, and FP (FN) represents number of false positive (false negative) classified pathological images.

### 5.1. Comparison of the Accuracy with Previous Methods

To verify the effectiveness of the method, we conduct comprehensive comparative experiments. For the three-class classifications, our method achieved 93.4% average accuracy based on the PathoIDCG dataset (see Table 2). The morphological differences between grade 1 (G1) and grade 2 (G2), as well as grade 2 (G2) and grade 3 (G3), is very subtle, so it is difficult to distinguish. This problem is reflected by our experimental results and previous studies. For this reason, previous studies have only focused on the classification tasks of G1 and G3. We have made comprehensive comparisons with previous state-of-the-art studies and the classic CNN: ResNet50 [37] and Xception [36]; the experimental results are shown in Table 2. It can be seen from the results that our method has achieved good classification accuracy in each category. However, only 94.1% and 93.9% accuracy are achieved on G1 vs. G2 and G2 vs. G3, respectively. Compared with the classification results of these two difficult categories, the classification accuracy of G1 vs. G3 is much better, reaching 97.8%.

### 5.2. Confusion Matrix and AUC

**Figure 7:**
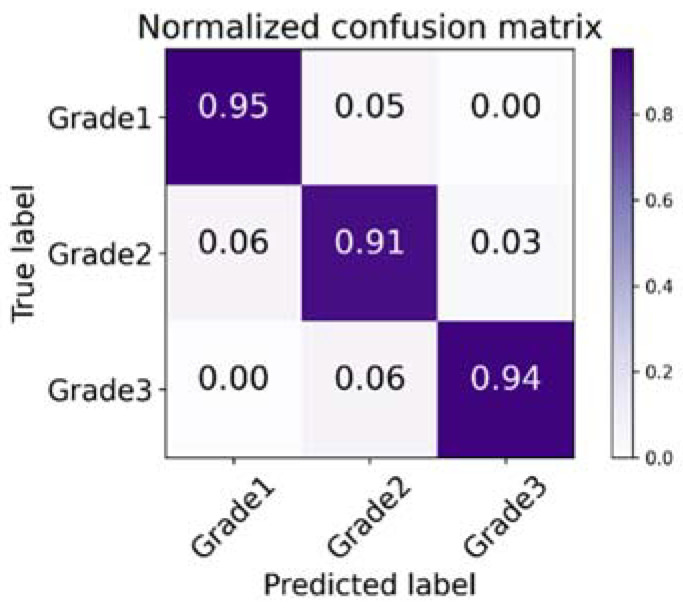
Visualization of normalized confusion matrix.

**Figure 8:**
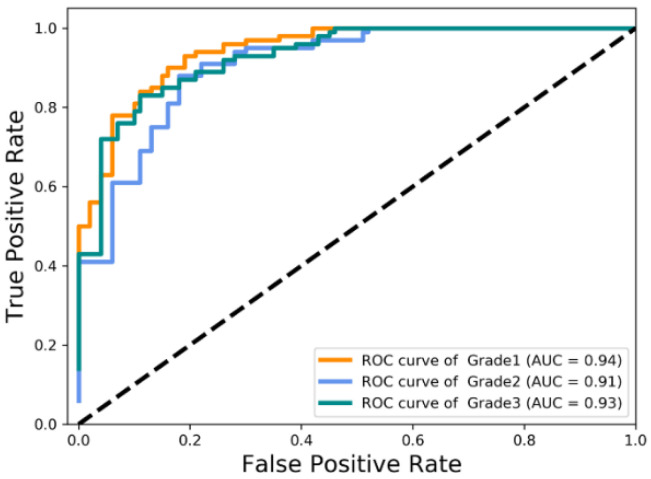
Visualization of receiver operating characteristic curve (ROC) and area under curve (AUC).

We conduct experiments on the PathoIDCG dataset to comprehensively evaluate the performance of our method. The confusion matrix of the predictions is presented in Figure 7 by using the proposed NGNet on the test set. Figure 8 shows the mean area under curve (AUC) of 0.93, corresponding to 0.94, 0.91, and 0.93 based on receiver operating characteristic analysis.

As seen from the experimental results in Figure 7 and Figure 8, the results obtained in G1 vs. G2 and G2 vs. G3 are not as good as the classification results of G1 vs. G3. This also further illustrates that the classification bottleneck is to learn more distinguished features for similar categories.

### 5.3. Nuclei Segmentation Results

**Figure 9:**
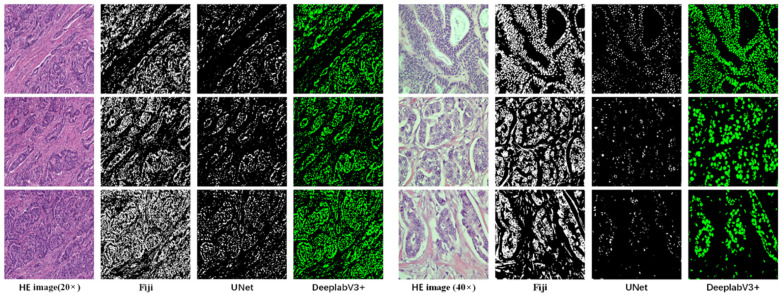
Nuclei segmentation results using Fiji (Watershed), UNet, and DeepLabV3+ (proposed). The left three rows are comparisons of the segmentation results at 20× magnification, and the right three rows are comparisons of the segmentation results at 40× magnification.

To select the suitable method for nucleus segmentation, we compare with three methods: Watershed, UNet [38], and DeepLabV3+ [35]. The watershed is the most representative traditional image processing method, and the version we used in the experiment is Fiji [39]. At the same time, we also conduct experiments on representative deep-learning methods UNet [38] and DeepLabV3+. As can be seen from Figure 9, DeepLabV3+ is suitable for our cell nucleus segmentation task, and achieved satisfactory results.

We perform a visual qualitative analysis of the segmentation results only. The visual display of the segmentation results is shown in Figure 9. Because we do not have the ground truth of nuclei segmentation for the PathoIDCG dataset, we did not use traditional quantitative indicators such as mean intersection over union (mIOU) to measure the segmentation effect. Our segmentation network is trained on the well-annotated dataset proposed by Kumar et al. [33]. After the segmentation network is well-trained, we directly use this trained segmentation network to segment the IDC grading dataset. Moreover, traditional metrics cannot measure the segmentation results we need. For example, we think that a slightly larger segmentation that includes the edge background of the nuclei may be better. However, the segmentation of nuclei containing a large number of missing nuclei is very poor.

### 5.4. Grad-CAM Visualization

Gradient-weighted class activation mapping (Grad-CAM) is a method proposed by Selvaraju et al. [40] to produce visual explanations (heat map) of decisions, making CNN-based methods more transparent and explainable. Grad-CAM can generate a rough location map to highlight important areas in the image for prediction. This method only considers the pixels and locations that have a positive impact on the classification result because we only care about the locations that have a positive impact on the classification.

**Figure 10:**
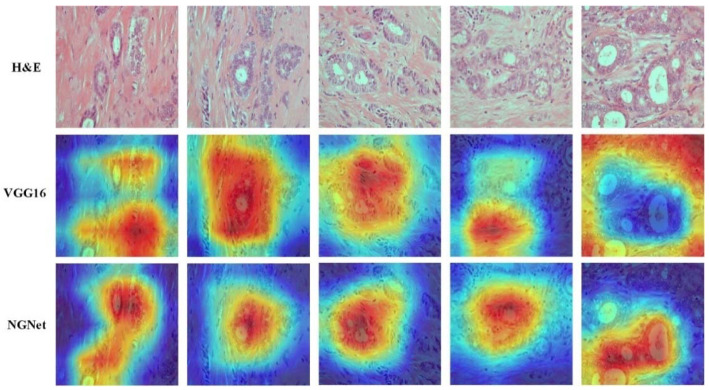
Visualization of class activation maps using Grad-CAM method. Red regions indicate a high score of a certain class. The first line is the pathological image. The second line and third line are the visual heat map using VGG16 and our proposed NGNet, respectively, as the backbone of Grad-CAM. Figure best viewed in color.

In this section, we use the Grad-CAM method to visualize the pathological image regions that provide support for a particular classification result. We compare the Grad-CAM experimental results of NGNet with VGG16, as shown in Figure 10. From the experimental results, it can be found that the experimental results of NGNet are more focused on the area related to the nuclei. Moreover, NGNet can further refine the nuclei-related feature representations. As shown in the pathological image and the corresponding heat map in Figure 10, attention not only focuses on the nuclei-related area but also focuses on the gland-related nucleus area. This is consistent with the medical knowledge of NGS. Clinically, breast cancer grading is adopted by pathologists through NGS, and one of the key evaluation criteria is the formation of glands.

### 5.5. Ablation Study

To evaluate the effectiveness of each component in our proposed method, we conducted an ablation study. The experimental results on the test set are shown in Table 3. The hyperparameters of the experiment include the following: the loss function is categorical cross-entropy, the learning rate is 0.00002, the optimizer is RMSProp, and a total of 300 epoch iterations are performed.

We conduct comparative experiments on accuracy, sensitivity, specificity and AUC. First, because our single branch network structure is similar to VGG16, we compare the classification performance of NGNet and VGG16. The experimental results show that NGNet has achieved much better results than just using VGG16. Then, we compare the experimental results of NGNet with different experimental configurations. NGNet has achieved better results even with a simple fusion of pathological images and nuclear images; that is NGNet without nuclei-guided attention (NGA) and nuclei-guided fusion (NGF) module. After adding the NGA module and NGF module to NGNet, the best results are achieved. Specifically, compared with NGNet without NGA and NGF module, NGA and NGF module bring an AUC improvement of 0.01 and 0.03 to the network, respectively. When using the NGA and NGF module at the same time—that is, our proposed NGNet—it brings an AUC improvement of 0.04 to the network. The experimental results fully demonstrate the advantages of NGA module and NGF module in NGNet, and also demonstrate that each module is indispensable. The experimental results are shown in Table 3.

## 6. Conclusions

In this paper, the proposed NGNet can ensure that the network is focused on nuclei-related features, so as to learn fine-grained feature representations for breast IDC grading. Through extensive experimental comparisons, it was shown that NGNet outperforms the state-of-the-art method and has the potential to assist pathologists in breast IDC grading diagnosis. In addition, we released a new dataset containing 3644 pathological images with different magnifications (20× and 40×) for evaluating breast IDC grading methods. Compared with the previous publicly available dataset of breast cancer grading with only 300 images in total, our number of images is an order of magnitude greater. Therefore, the dataset can be used as a benchmark to facilitate a broader study of the breast IDC grading method.

In future work, to further improve the classification performance of breast IDC grading, medical knowledge embedding and semi-supervised learning are two promising directions. Whether in the field of natural image analysis or medical image analysis, the research on the network structure of deep learning has been very comprehensive. Therefore, only by improving the network structure to further improve the classification performance is limited. There are few studies on how to combine medical knowledge with pathological image to further improve classification performance [41]. If we can embed medical knowledge in the end-to-end network learning, the performance of the IDC grading method will be further improved. In terms of pathological image datasets for IDC grading, it is impractical to label a sufficiently large dataset because the cost of labeling pathological images is high. However, the amount of unlabeled pathological image data in each hospital is very large [42]. If a small labeled dataset and a large unlabeled dataset can be used at the same time, the performance of the IDC grading method may be further improved to a level that can be used clinically.

## Figures and Tables

**Table 1:** The overall description of the PathoIDCG dataset.

Description	Value
No. pathological images (total)	3644
No. pathological images (40×)	1158 (361 G1, 480 G2, 317 G3)
No. pathological images (20×)	2486 (600 G1, 641 G2, 1245 G3)
Size of pathological images	1000 × 1000 pixels
Magnification of pathological images	20×, 40×
Color model of pathological images	R(ed)G(reen)B(lue)
Memory space of pathological images	~1 MB
Type of image label	Image-wise

**Table 2:** Comparison of accuracy with previous methods.

Methods	Acc (%) G1 vs. G2	Acc (%) G1 vs. G3	Acc (%) G2 vs. G3	Acc (%) G1 vs. G2 vs. G3
Naik et al. [15]	-	80.5	-	-
Doyle et al. [14]	-	93.0	-	-
Basavanhally et al. [16]	74.0	91.0	75.0	-
Wan et al. [17]	77.0	92.0	76.0	69.0
ResNet50 [37]	87.5	91.0	88.5	87.2
Xception [36]	88.3	92.3	88.6	87.9
NGNet	94.1	97.8	93.9	93.4

**Table 3:** Ablation study results with different configurations on the test set.

Methods	Acc.	Sensitivity	Specificity	AUC
VGGNet (pathology image only)	85.1%	86.0%	85.3%	0.87
VGGNet (nuclei image only)	80.6%	81.2%	79.2%	0.79
NGNet (w/o NGA and NGF)	90.6%	89.3%	89.8%	0.89
NGNet (w/o NGF)	92.2%	93.8%	91.1%	0.92
NGNet (w/o NGA)	91.8%	91.6%	90.9%	0.90
NGNet (proposed)	93.4%	95.3%	92.9%	0.93

## Data Availability

The dataset is publicly available from https://github.com/YANRUI121/Breast-cancer-grading (accessed on 1 April 2022).
